# Status of surface modification techniques for artificial hip implants

**DOI:** 10.1080/14686996.2016.1240575

**Published:** 2016-11-25

**Authors:** Subir Ghosh, Sylvester Abanteriba

**Affiliations:** ^a^ School of Engineering, RMIT University, Melbourne, VIC, Australia

**Keywords:** Surface modification, texturing, coating, grafting, hip joint, 30 Bio-inspired and biomedical materials, 212 Surface and interfaces, 306 Thin film / Coatings

## Abstract

Surface modification techniques have been developed significantly in the last couple of decades for enhanced tribological performance of artificial hip implants. Surface modification techniques improve biological, chemical and mechanical properties of implant surfaces. Some of the most effective techniques, namely surface texturing, surface coating, and surface grafting, are applied to reduce the friction and wear of artificial implants. This article reviews the status of the developments of surface modification techniques and their effects on commonly used artificial joint implants. This study focused only on artificial hip joint prostheses research of the last 10 years. A total of 27 articles were critically reviewed and categorized according to surface modification technique. The literature reveals that modified surfaces exhibit reduced friction and enhanced wear resistance of the contact surfaces. However, the wear rates are still noticeable in case of surface texturing and surface coating. The associated vortex flow aids to release entrapped wear debris and thus increase the wear particles generation in case of textured surfaces. The earlier delamination of coating materials due to poor adhesion and graphitization transformation has limited the use of coating techniques. Moreover, the produced wear debris has adverse effects on biological fluid. Conversely, the surface grafting technique provides phospholipid like layer that exhibited lower friction and almost zero wear rates even after a longer period of friction and wear test. The findings suggest that further investigations are required to identify the role of surface grafting on film formation and heat resistance ability under physiological hip joint conditions for improved performance and longevity of hip implants.

## Graphical abstract

**Figure:**
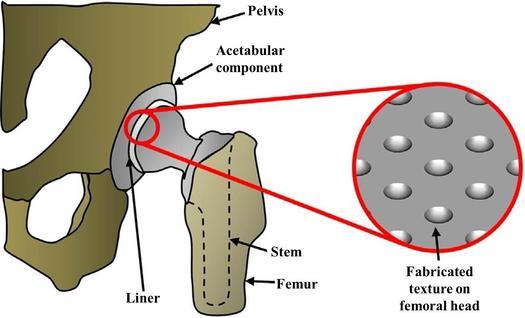

## 1. Introduction

Total hip arthroplasty (THA) is one of the most successful treatments for patients with severe hip osteoarthritis and rheumatoid arthritis. This treatment has improved the quality of life by introducing a solution to the affected joints. The demand of THA treatments has been increasing steadily because of the rise in the population of the elderly.[1] Approximately one of every four adults is affected by arthritis in the USA.[2] According to Deloitte Access Economics, current trends suggest that 7 million Australians will suffer from some form of arthritis by 2050.[3] Improvements in THA treatments are needed due to implant wear, dislocation, implant fracture, and aseptic loosening. However, aseptic loosening resulting in periprosthetic osteolysis remains a serious problem, which degrades prosthetic joint survival and clinical success. Up to 20% of patients implanted with conventional polyethylene (PE) suffer from aseptic loosening within 10 years of implantation and some of them become disabled due to pain and lack of functionality.[4] The only therapeutic action is revision surgery. The number of revision surgery has been increasing and is estimated to be twice by the year 2026.[5] The revision rate would be higher for the number of younger or adult patients due to their higher life expectancy.[5] This would lead to a cumulative social and economic impact if preventive mechanisms are not successfully implemented. High friction and consequent wear of artificial hip implants after 10–15 years of implantation are the major issues leading to revision surgery.[6,7] Therefore, surface modification techniques can be developed to improve the implant quality considering the lifespan of younger patients.

Various surface modification techniques have been developed so far to engender longer life of joint prostheses, including surface texturing (dimpling) and surface coatings.[8,9] Surface modification techniques are generally applied on femoral head surfaces. Surface texturing improves tribological performance. It increases the film thickness between the mating components by acting as a lubricant reservoir. This film thickness provides additional lift effect by generating hydrodynamic pressure between the converging surfaces. Thus, it protects the surfaces from coming into contact and therefore prevents the generation of solid friction. The dimples produced by surface texturing can trap wear debris in boundary lubricating conditions. Further, it decreases the contact area and thereby reduces adhesion. Roy et al*.* [6] reported nearly 22% less friction and 53% wear reduction for textured surfaces compared to non-textured surfaces while texturing on disc surfaces. They also showed that dimple density and depth have significant influences on improving the tribological performance. Ghosh et al*.* [10] also found similar results and comparatively better wear resistance provided by the textured surfaces under body oriented fluids to water lubricants. Further investigations of a combination of texturing and coating techniques have reported improved wear performance.[11] Surface coating techniques have been used to engineer the bearing surfaces of artificial joints to provide a highly wear-resistant surface. Surface coating increases the mechanical properties of the contacting surface, enhancing the wear and corrosion resistance of the contact surfaces. The graphite wear products of coated surfaces form a protecting layer on the counterparts, and thus act as a solid lubricant. Choudhury et al*.* [11] reported superior tribological results with diamond-like-carbon (DLC)/polyethylene sliding pairs, whereas a-C:N provided the finest performance at DLC/DLC pairs. Another surface modification technique known as surface grafting on acetabular liner has significant influence on friction and wear reduction for joint implants.[12] It possesses an excellent biocompatibility and anti-biofouling ability,[13] provides unique surface properties of high lubricity, low friction, anti-protein adsorption and cell adhesion resistance,[14] and forms a hydrated lubricating layer and hence increased wear resistance.[15] The grafted surface for other medical devices inhibited biological reactions when they were in contact with living organisms, and are now clinically used on the surfaces of intravascular stents, soft contact lenses, and artificial lungs and hearts under the authorization of the US Food and Drug Administration.[15] It is recognized that a nanometre scaled phospholipid layer covers articular cartilage that protects the articulating surface from mechanical wear, and facilitates a smooth motion of the joint during daily activities.[13] In addition, the grafted particles are biologically inert and do not cause consequent bone-resorptive responses, indicating that this technique prevents wear particles production and biological reactions to such particles in THA.[16]

Though advances in implant design and materials have improved the quality of life for many people, there is a considerable room for further developments. This review discusses and collates recent findings on detailed surface modification techniques for artificial hip implants. The overview illustrates the tribological aspect of artificial hip interfaces as well as the commonly used implants and their merits and demerits based on useful life and functionality of artificial implants, both *in vivo* and *in vitro* conditions. The aim of this study is to identify the most acceptable surface modifications technique for longevity of artificial hip implants.

## 2. Artificial hip joint prostheses

According to the National Joint Replacement annual report in 2015, published by Australian Orthopaedic Association, 43,183 hip replacements were reported to the registry in 2014; 6.3% more than in 2013.[3] The number of primary total hip replacement due to severe arthritis has increased by 72.5% compared to 2003.[3] The revision in replacement surgery is a major problem in THA treatments. The revision rate was an increase of 25.1% compared to 2003. However, the revision burden has been decreased by 2.4% in last four years. The main reason behind the decrease in revision rate is due to the implementation of larger size femoral head. It is found that femoral head size equal to or larger than 32 mm is effective in reducing the revision rate. The most common reasons for revision are loosening/lysis (47.8%), prosthesis dislocation (14.1%), infection (14.1%), and fracture (10.4%).[3] Loosening and lysis can be caused by inflammatory reactions due to the production of small wear particles. The higher friction and consequently wear have a significant effect on the movement of the femoral head. Movement of the femoral head causes prosthetic dislocation. Hence, minimization of friction and wear is desirable to prevent revision surgery of artificial hip implants. This section will describe different artificial joint interfaces. Commonly used material combinations for hip prostheses are: (1) metal on polyethylene (MoP); (2) ceramic on polyethylene (CoP); (3) ceramic on ceramic (CoC); (4) ceramic on metal (CoM); (5) metal on ceramic (MoC); and (6) metal on metal (MoM). Generally, two types of femoral head (metal and ceramic) and three types of acetabular cup (metal, ceramic, and polyethylene) are used in combination as the bearing surface for artificial hip implants.

### 2.1. MoP hip implants

The most common THA material combination has been a metal femoral head paired with polyethylene acetabular cup (MoP implants). Different types of metal are used as femoral component, e.g. titanium (Ti), Ti alloys, Co–Cr–Mo and stainless steel. The use of austenitic stainless steel has been limited due to its poor wear resistance properties. Ti alloys and Co–Cr–Mo are frequently used in THA. A comparative study showed that the highest linear wear was about 1 µm for Ti-6Al-4 V alloys followed by stainless steel (0.2 µm) and Co–Cr–Mo (0.1 µm) after one million cycles.[17] Polyethylene as acetabular cup has been a leading material for the last 30 years because of its low friction coefficient with the metallic counterparts. Recently, a newer highly cross-linked polyethyelene (CLPE) has been developed that possesses improved wear resistance properties with enhanced mechanical properties.[18] It is irradiated with a high-dose (∼100 kGy) gamma-ray or electron beam. Cross-linking reduces the degree of molecular orientation, and thus improves the wear resistance.[19] However, the polyethylene counterparts suffer maximum wear against hard metal femoral heads in MoP hip implants. The generated wear debris mixture in the lubricant further increase the wear rate. Likewise, the produced wear particles enter into the periprosthetic tissues to react with macrophages and giant cells. Thereafter, the macrophages release pro-inflammatory cytokines that leads to osteolysis and subsequent loosening of the prostheses.

### 2.2. CoP hip implants

Ceramic and polyethylene are now a promising implant pair, because both materials exhibit low friction coefficient, and consequently less wear. CoP implants have better wettability properties than MoP implants. Ceramic materials currently used as hip implants include alumina (Al_2_O_3_), zirconia (ZrO_2_), and alumina–zirconia composite. Alumina ceramic has excellent corrosion resistance, good biocompatibility, high strength and effective wear resistance. The major advantage of alumina materials in implant application is their highly polished (±10 nm) surface, which leads to low friction and wear. However, alumina has low fracture toughness which is a reason for early failure of CoP implants. The failure of alumina due to its high brittleness and slow crack growth can be minimized by the use of zirconia and alumina composite material. Zirconia is exceedingly hard with good mechanical properties that make it very useful as a biomaterial. The clinical success of alumina bearing materials under less mechanical load was reported in follow-up studies.[20] Hip joints maintain very low load, hence alumina is effective as a hip implant material.

### 2.3. CoC hip implants

CoC implants have good longevity due to their low friction and wear. Alumina on alumina, and zirconia on alumina are generally used as prosthesis pairs. Villermaux [21] reported extensively low wear at 0.1 mm^3^ per million cycles for zirconia femoral heads paired with alumina; this is significantly lower compared to other hip implants. However, one of the major problems associated with CoC implants is squeaking. This happens because of direct contact of two hard surfaces. Squeaking is found to have significant influence on wear mechanism of CoC hip implants.[22] It also creates vibration in the system during sliding or rolling action.

The fracture toughness of zirconia is about two times greater than alumina. This increases the crack propagation resistance.[23] It also possesses higher bending strength. Thus, zirconia has led to new types of implant design. But zirconia exhibits a progressive ageing degradation in the presence of fluids. Considering the limitation of earlier developed ceramics, a zirconia-alumina composite could provide better properties. It was found that a material composite combination of 80% tetragonal zirconia polycrystals and 20% alumina exhibits outstanding mechanical and tribological performance.[24] Besides the improved friction and wear performance by CoC implants, the stress shielding effect has limited their application in hip implants.

### 2.4. CoM and MoC hip implants

Ceramic heads–metal cups and metal heads–ceramic cups (CoM and MoC hip implants, respectively) are used in a small number of THA. Due to the higher wear rate of polyethylene, interest in other materials has grown as alternative bearings for hip implants. Ceramic is comparatively harder than metal substrates. As a result, the wear rate is higher for metals when ceramic acts as a counterpart. MoC implants have more friction and more wear than CoM implants due to crucial influence of applied loads on the friction. When the femoral metal head faces higher loads and ceramic counterparts move over them, they release more metal ions from the surface. However, the tribological performance of MoC and CoM implants are found to be similar for larger femoral heads.

### 2.5. MoM hip implants

Interest in MoM hip implants had increased in past decades due to their improved wear resistance properties. Moreover, the metal possesses excellent mechanical properties, electrical and thermal conductivity. However, THA procedures using metal on metal bearings are now rarely carried out. Though MoM exhibits low wear volume, it contributes higher concentration of metal ions. Nanoscale size wear particles of the metals are believed to cause adverse local tissue reactions comprising metal hypersensitivity and allergic reactions.[25] They cause pseudo-tumours, large effusions and/or periprosthetic bone resorption.[25] Increased metal ion concentrations in organs results in osteolysis, pain, and corresponding wear and failure of the implant.[26] Co–Cr–Mo releases Cr, Mo and Ni metal ions and forms a passive oxide layer in the human body environment.[27] The corrosion products of Co–Cr–Mo have been identified as more toxic to human body.[28] Therefore, Ti alloys are considered more reliable for hip implants. However, toxicity of metal bearings could be minimized by surface modification techniques.

## 3. Surface modification techniques and their recent developments

Surface modification of biomaterials has led to improvement in device multi-functionality, tribological and mechanical properties, and the biocompatibility of artificial devices, while avoiding the need for large development costs in terms of money and time. It changes the physiochemical properties of the materials such as surface charge, surface energy and surface composition. Optimal surface, physical and chemical properties could be achieved by altering the functionality of the bulk materials. Wear rates of the implants have been minimized by applying higher wear resistance materials *in vitro.* Researchers have developed new alloy materials in the last two decades that are able to resist wear. Nevertheless, the physiological condition *in vivo* is different from *in vitro* condition. As a result, success of the implant materials *in vitro* does not always bring success in clinical outcomes. It has been observed that highly resistant materials do not always perform well under body fluids. To improve the implant quality and longevity of the implants, friction and wear reduction is the best solution. The surface modification technique is becoming an increasingly popular method due to its improved performance in implant application. However, wear is still inevitable. Hence, improvements in the surface modification technique are needed. The following section will provide a detailed description of different surface modification techniques for artificial hip implants. Recent studies and developments of the surface modification techniques will also be outlined.

### 3.1. Surface texturing

The surface texturing technique has been introduced in implant design to obtain the benefit of the lubricating effect. It was previously used on golf ball surfaces to improve their aerodynamic characteristics. Spiral grooves were produced on bearings to build up pressure in order to separate the bearing surfaces. Surface texturing is now employed in tribology, optics, physics, energetics, biomedicine, electronics and metrology.

**Figure 1.**
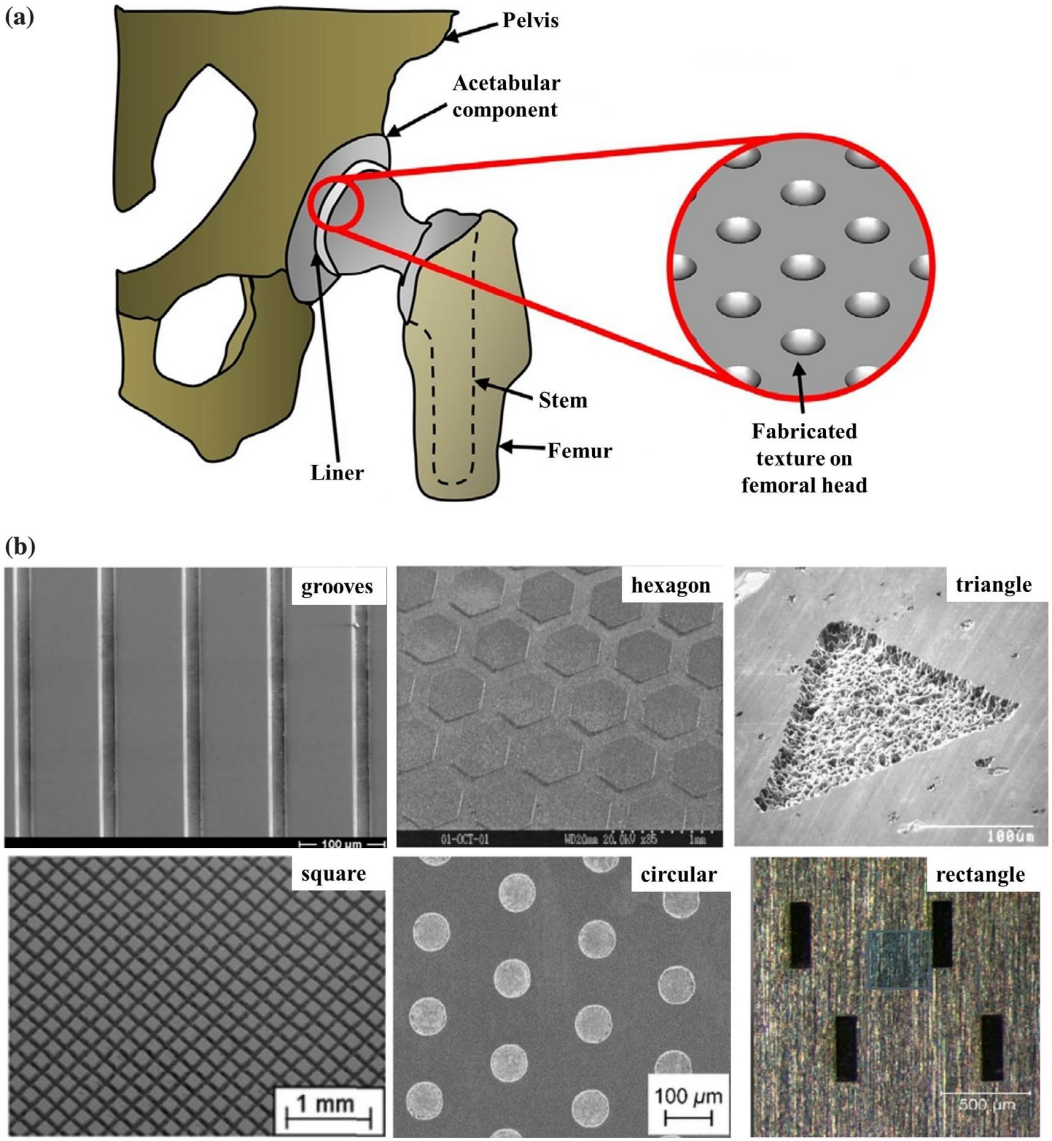
(a) Surface texturing on femoral head [33] and (b) SEM images of different types of fabricated surface texturing.[9]

This technique is popular in implant design due to the improved friction and tribological performances. It produces micro-textures on implant surfaces that have a number of benefits over smooth surfaces: (1) they act as a lubricant reservoir;[29] (2) they increase hydrodynamic pressure under sliding conditions;[30] (3) they store the produced wear debris or foreign materials in dimples;[31] (4) they decrease the contact area;[32] and (5) they minimize friction and wear.[7] Textured surfaces are more effective, especially in boundary and elastohydrodynamic regimes. Micro-dimple functionality is initially confirmed by correct optimization of its geometrical parameters, still the influence of dimples quality is not avoidable. Figure 1(a) and (b) show the fabricated surface texturing on articulating femoral head and scanning electron microscopy (SEM) images of different types of fabricated textured surfaces.

#### 3.1.1. Recent studies of surface texturing for improved tribological performance

The tribological performance of the implant surface does not depend only on the surface texturing technique but also on the geometrical parameters such as dimple diameter, dimple depth, dimple shape, dimple pattern and dimple density.[34] Roy et al*.* [6] revealed that dimple diameter of 400 μm with 15% density showed the enhanced friction and wear performance compared to other geometries (diameter: 300 or 400 μm and density: 5 or 10%). They revealed that larger sized dimple with higher density reduced the friction and wear. Conversely, Huang and Wang [35] reported enhanced lubricating effect of the smaller size dimple with high pore density due to the uniform distribution of lubricant in micro-dimples. This also confirms the role of dimples as lubricant reservoirs. The lubricant squeezes out from dimples to minimize the sliding contact under loading conditions. However, surface roughness could be increased with dimple density. As a result, surface defects possibly increased due to the production of more micro-dimples. There is a chance of high friction and fatigue wear if the surface defects increase the subsurface stress. Kaneta [36] reported the reduction in local film thickness due to the increase in dimple depth. Thus, the low film thickness led to high friction coefficient and consequent wear. Circular shaped dimples are commonly applied because they can be easily fabricated with high precision. Ito et al*.* [37] investigated the tribological performance of a circular textured Co–Cr–Mo surface and observed 17% reduction in friction and 36% polyethylene wear reduction. Regular shapes such as ellipses with round or curved edges were observed to minimize the friction and to enhance load carrying capacity considerably compared to other shapes. On the other hand, Shen and Khonsari [38] reported significant improvement of the tribological performance by applying trapezoidal-like shape during bidirectional hydrodynamic sliding. Researchers are trying to optimise the geometries of dimples using ordinary shape. Unfortunately, no unique approach had been developed so far in view of geometries of dimples to reduce friction and wear. Different researchers suggested different optimum geometrical characteristics. Still, these do not minimize the gap of understanding the influence of surface texturing for better tribological performances. The operating conditions are also influential factors to optimize the surface texturing. The hydrodynamic pressure is increased at high speed and low load conditions where maximum load is carried out by operating fluid. Surface texturing provides additional lift due to the converging film thickness, generating hydrodynamic pressure, whereas the surfaces do not come in contact. The ability of bearing the load increases with film pressure. In contrast, maximum load is carried out by the textured surface under high load and low speed condition when the dimpled surface acts as a lubricant reservoir. Interestingly, Yan et al*.* [39] reported that dimple density is the dominant factor independent of the different operating conditions. However, Ghosh et al*.* [10] revealed that textured surfaces exhibited higher friction under higher load because textured surfaces were not able to resist that higher load. Textured surfaces produce wear particles that act as a third body in the wear mechanism. Nevertheless, the textured surface showed improved tribological performance under simulated body fluid (SBF) compared to water lubricant, even at higher load. Choudhury et al*.* [40] reported a better load bearing capability of a textured surface in hip simulator study. They showed that the dimpled surface exhibits higher film thickness compared to a flat surface. Thus, load is supported by the fluid hydrodynamic pressure. Table 1 summarizes the recent tribological studies of artificial implants in the last 10 years, to identify the effect of surface texturing for improved friction and wear performance. It indicates that laser surface texturing (LST) and computer numerical controlled (CNC) machining techniques are more popular for dimple fabrication. A significant reduction in friction and wear with textured surfaces was observed in most literature reviewed. However, the applied load was different in different studies. Hence, it is difficult to optimize the surface texturing by evaluating friction and wear rate under different loading conditions. Few researchers suggested incorporating coating technique with texturing because thin film coatings on textured surface provide very low friction.[10,40] It minimizes the problem associated with initially high friction for textured surface in line contact that resulted from low contact width and wedge angle.[41,42] Recent studies revealed that DLC coated textured surface enhanced wear resistance properties of implant materials.[10,43,44] He et al*.* [43] observed that DLC coated textured surfaces with 24% dimple density exhibited higher friction but lower wear rate than those with 44% dimple density. However, both of them provided improved wear rate than a smooth DLC coated surface, but higher friction. It could be concluded that lower friction is not always essential to reduce the wear rate. Choudhury et al*.* [11] showed that enhanced friction and wear performance were independent on a textured surface. They concluded that DLC coated surfaces exhibited the lowest friction coefﬁcient and it was nearly same for the dimpled and the non-dimpled surface. Choudhury et al*.* [40] concluded in another study that DLC coated dimpled surface has dual benefits: a DLC coated surface reduces the friction coefficient, whereas a textured surface improves the lubricating effect by providing hydrodynamic lift between the sliding contact.

### 3.2. Surface coating

Improved wear resistant of artificial implants may be accomplished by the application of surface coatings. Surface coating provides morphological (topographical design), physiochemical (changes to surface energy, charge, or composition), and biochemical adaptations (how the cells react) to existing bulk materials. Surface coatings reduce adverse cellular response caused by the generated wear particles. Thus, it engenders useful life of artificial implant. Various hard and wear resistant coatings such as metal nitrides, carbides, carbonitrides, and DLC are used in artificial implant applications. These coatings enhance the mechanical and physical properties such as hardness, elastic strain and wettability of the surface. The improved wettability of the surface leads to the low friction and consequently the low wear of implant materials. Surface coating protects a bulk surface from tribo-corrosion.[47,48] The performance of the coated surface also depends on the coating deposition technique. An ideal deposition process can provide quality surface such as a dense homogenous coated surface with excellent adhesion to the substrate.

#### 3.2.1. Recent studies of surface coating for improved tribological performance

**Figure 2.**
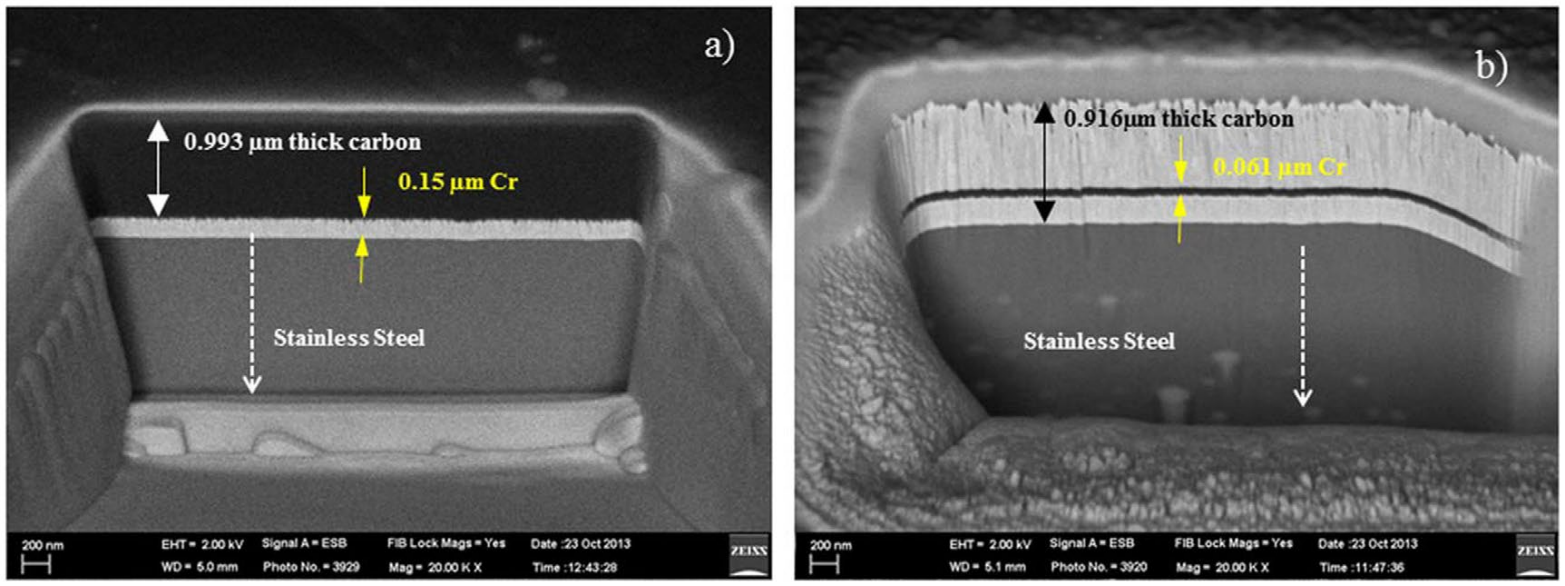
Cross section images of deposited (a) a-C:H and (b) Ta-C on stainless steel.[11]

Many attempts have been made to minimize the friction and the wear of hip implants. Though metal on metal hip bearings has reduced the linear wear about 40 times and volumetric wear about 200 times compared to metal on ultra-high molecular weight polyethylenes (UHMWPE),[49] the toxicity of metallic or UHMWPE debris has limited their application in hip implants. Therefore, surface coatings have been introduced to avoid the adverse effect of metallic surfaces. The coated surface provides a protective layer on the metal surface and improves the mechanical properties of the surface. Surface coating reduces the surface roughness, and thus lowers friction and wear. It was pointed out that Ta coated on Co–Cr–Mo with 5–12 nm surface roughness showed lower wear rate in the range of 4–5 × 10^−7^ mm^3^ N^−1^ m^−1^,[50] whereas Ta coated on Co-Cr-Mo surface with a surface roughness of 40 nm showed higher wear rate in the range of 0.755–1.249 × 10^−4^ mm^3^ N^−1^ m^−1^.[51] Moreover, TiN coating with higher surface roughness of 169 nm exhibited the higher wear rate at 6 × 10^−4^ mm^3^ N^−1^ m^−1^. This suggests that lower surface roughness is essential to minimize the friction and the wear of hip implants. Low surface roughness leads to better surface wettability.[52] The surfaces with better wettability enhance the lubrication and perform well even in the absence of lubricant. DLC coatings have better wettability compared to graphite-like carbon (GLC), Ta and TiN coatings. Table 2 summarizes tribological studies of artificial implants in the last 10 years to identify the effect of surface coatings for improved friction and wear performance. It can be seen that there is no standardization in the selection of experimental set-up, such as working load, contact pressure, frequency, testing lubricant, coating technique, coating thickness and type of simulator. It is thus difficult to compare friction and wear results between different studies. However, significant reductions in friction and wear value are obtained by the surface coating technique, whereas the DLC coating is more popular. The magnetron sputtering method has been widely used in recent tribological studies. Wang et al*.* [53] pointed out that CrN has better corrosion and wear resistance ability compared to TiN, TiA1 N coatings under synovial body fluid. However, DLC coating is superior in tribological performance in present of body fluid. The DLC (a-C:H /Ta-C) films significantly improved the surface hardness [11]. Figure 2 shows cross section SEM images of deposited (a) a-C:H and (b)Ta-C DLC films on stainless steel substrate.

**Table 1:** Summary of tribological studies on surface texturing.

Abbreviations: EDT stands for electrical discharge texturing, PVD for physical vapour deposition, SS for stainless steel, Ta-C for tetrahedral amorphous carbon and UHMWPE for ultrahigh molecular weight polyethylene.

**Figure 3.**
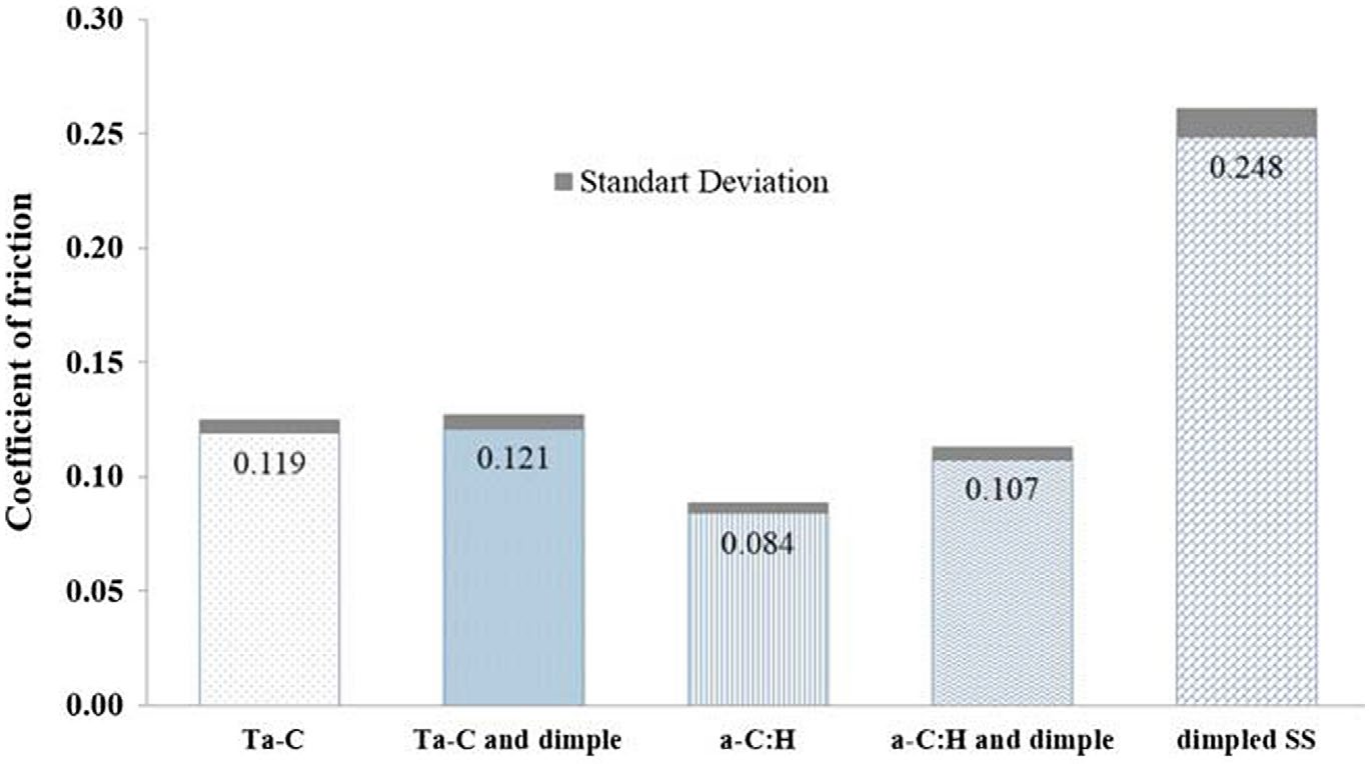
Friction coefficient results for different prosthesis heads.[11]

Cr was used as an interlayer in both cases that increased adhesive strength allowing stronger bonding between the steel and the carbon layers.[60] It has been suggested that multilayer coatings could improve the wear resistance of implant surface.[40,48,58] The intermediate layers such as chromium nitride, DLC, and TiN increase the hardness of the implant surface. Moreover, CrN increases the bonding strength between the coated material and the substrate.[61] The UHMWPE is favourable as a top layer for its better wear resistance properties compared to other polymers.[62] A coating thickness of more than 1 μm including an interlayer may be beneficial because very thin DLC layers produce pinholes during sliding.[8] The working lubricant can penetrate through these pinholes and thus increase the corrosion rate. The thick DLC film associated with interlayers will protect the coated surface from corrosion. The adhesion of DLC coated surfaces can also be improved by changing the substrate surface preparation, introducing interlayers such as CrN and N^+^ ion implantation or changing the DLC deposition parameters. DLC associated with CrN provided the lowest friction because it increased the corrosion resistance and adhesion of the coated layers.[53] However, sometimes a slow crack advancement or interlayer dissolution results in delayed delamination in corrosive media such as body fluid, although they show good mechanical adhesion during normal delamination tests.[63] Ghosh et al*.* [10] reported a significant reduction in friction and wear for a DLC coated dimpled surface independent of lubricating environment; the dimpled surface acted as a lubricant and wear debris reservoir and the DLC coated surface improved the lubrication. Conversely, Choudhury et al*.* [11] found negligible difference of friction coefficient between the dimpled and the non-dimpled surfaces while a-C:H coated surface exhibited lowest friction coefficient. Figure 3 summarizes the friction coefficient results for different prosthesis heads.

**Figure 4.**
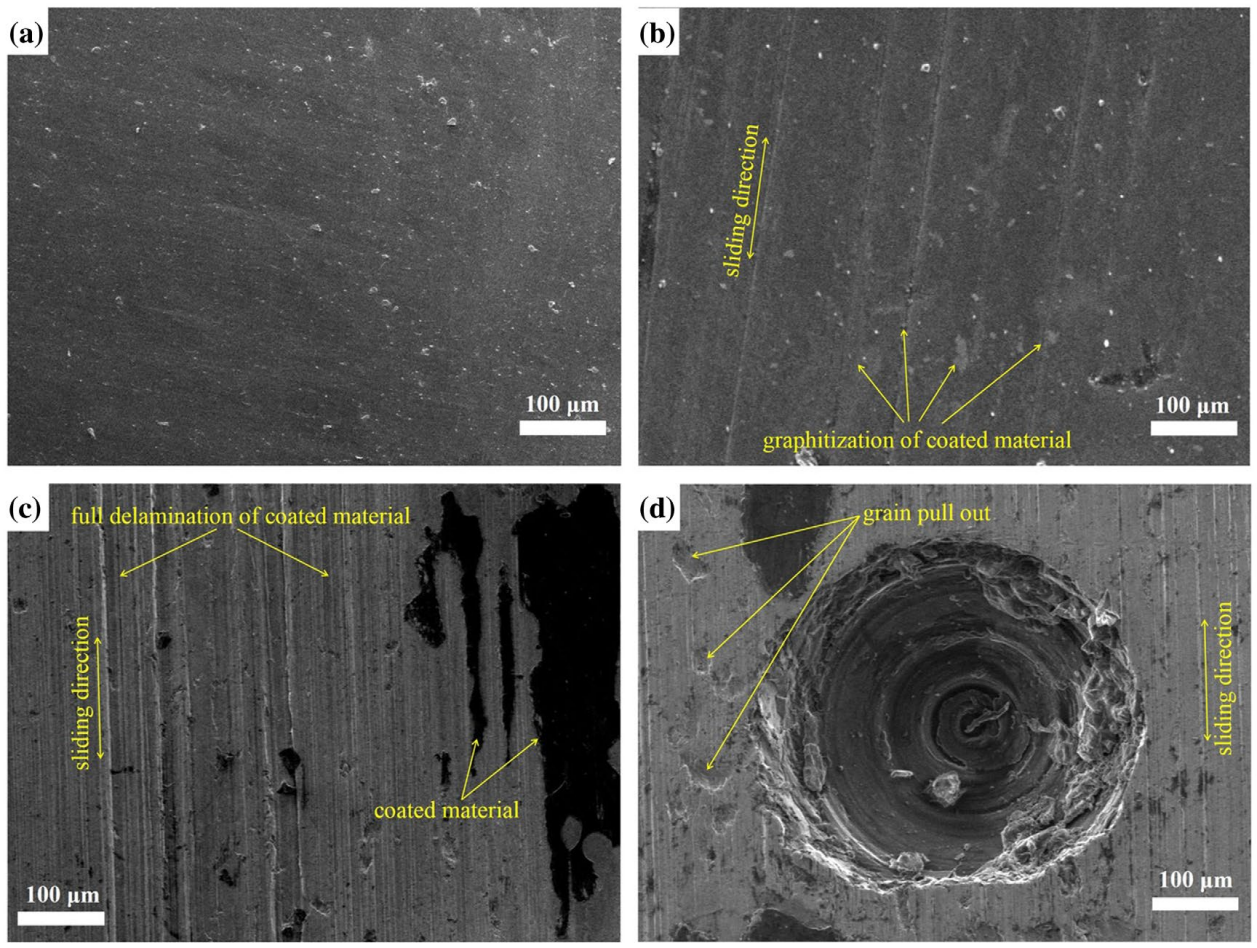
SEM images showing (a) as-deposited DLC-coated surface, (b) formation of film transfer due to load, (c) full delamination of coated materials at higher loads, and (d) wear track on dimpled area.[10]

A major drawback of DLC films is their early delamination during *in vitro* tests due to poor adhesion. The graphitization is caused after certain period of running the machine. The generated wear debris exacerbates wear rate. As a result, full delamination of the coated surface was observed at higher load. The grain pull-out on deposited surfaces were also observed attributed to the larger size wear particles. Figure 4 illustrates surface morphology of coated surfaces at different conditions.

Only a few clinical studies have been published on DLC coated bearing joints. They found that DLC coated surfaces failed *in vivo* due to crevice corrosion (CC) of the silicon based interlayer.[63,64] Instability of the interlayer towards the CC leads to the delamination of DLC coating. Hence, selection of interlayer is an important factor in reducing corrosion under corrosive media. Surface modification by DLC coating is not only good enough to resist wear of artificial joints but also well-designed structure of DLC coated surface with lower residual stress can provide the barrier for corrosion as well as delayed delamination *in vivo*.

### 3.3. Surface grafting

**Figure 5.**
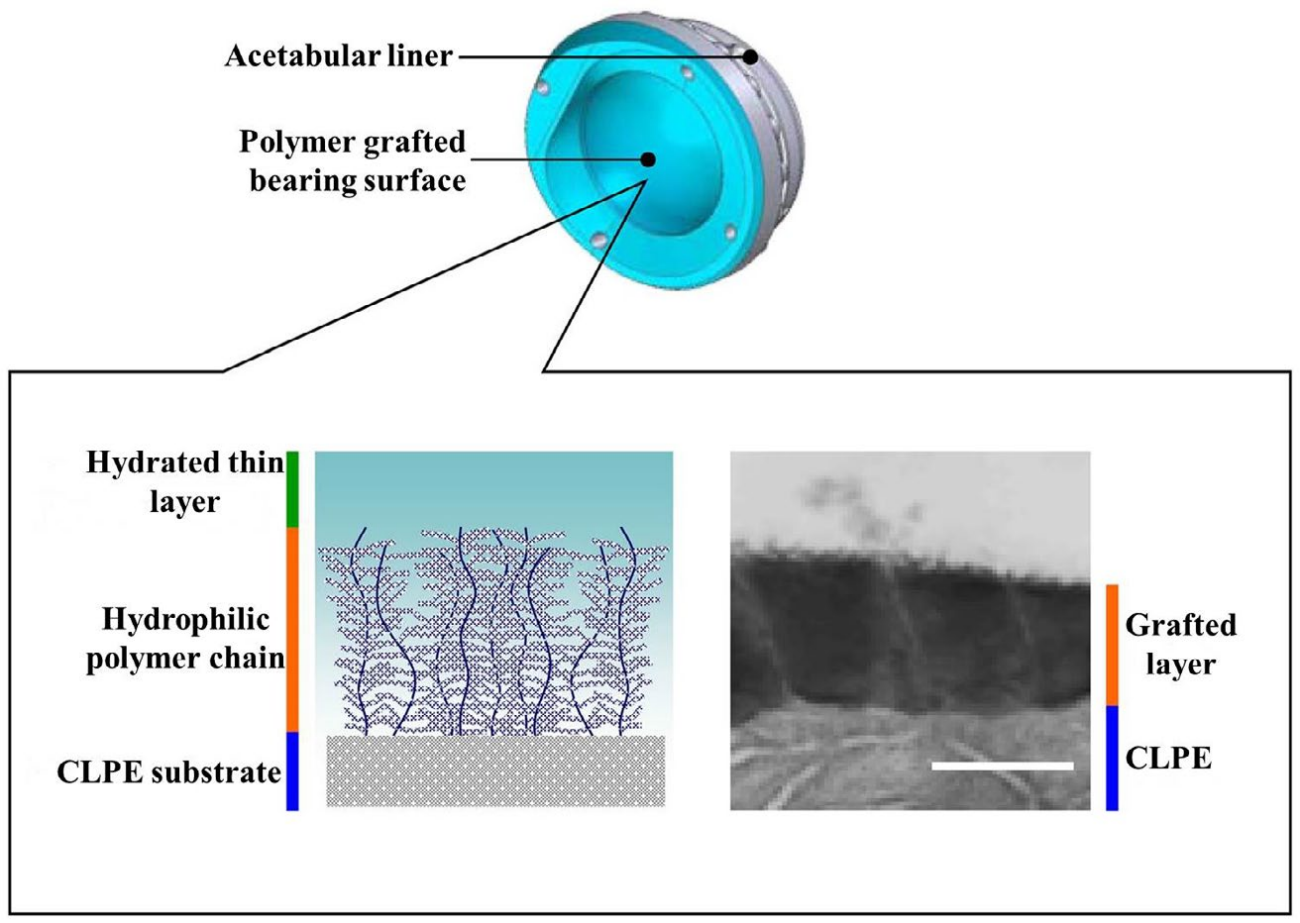
Schema of a THA with the PMPC-grafted CLPE liner. A transmission electron microscopy image of the surface is shown on the right. Orange and blue lines indicate the PMPC layer and the liner surface, respectively.[68]

The surface and bulk properties play a significant role in the success or failure of the implant devices. The surface properties such as wettability, chemical composition, softness/stiffness, porosity and roughness are key factors for the performance of a material in a range of biological environments.[65] Polymers are used as biomaterials. The bulk properties of polymers, such as conductivity, strength, polymer type, stiffness and general resistance to deterioration, are considered during their selection for biomedical applications.[66] The lower molecular weight proteins start adsorbing on implant surfaces after implantation of biomaterials in a biological environment. A high rate of protein adsorption may result in higher friction. However, protein adsorption depends on chemical composition, surface roughness and wettability of the surface. Hence, surface modification techniques such as polymer grafting are effective to avoid biofilm formation and protect the surface from biofouling. They also improve wettability of the surface and provide a more hydrophilic surface. The polymer surfaces possess a brush-like hydrophilic structure that is assumed to be similar to that of articular cartilage. The cartilage surface also has a the hydrophilic nature due to its water soluble macromolecules. These hydrophilic macromolecules stimulate the fluid film formation and reduce the friction of the joints.[7,67] Figure 5 shows the formation of hydrated thin layer on polymer grafted CLPE surface.

**Figure 6.**
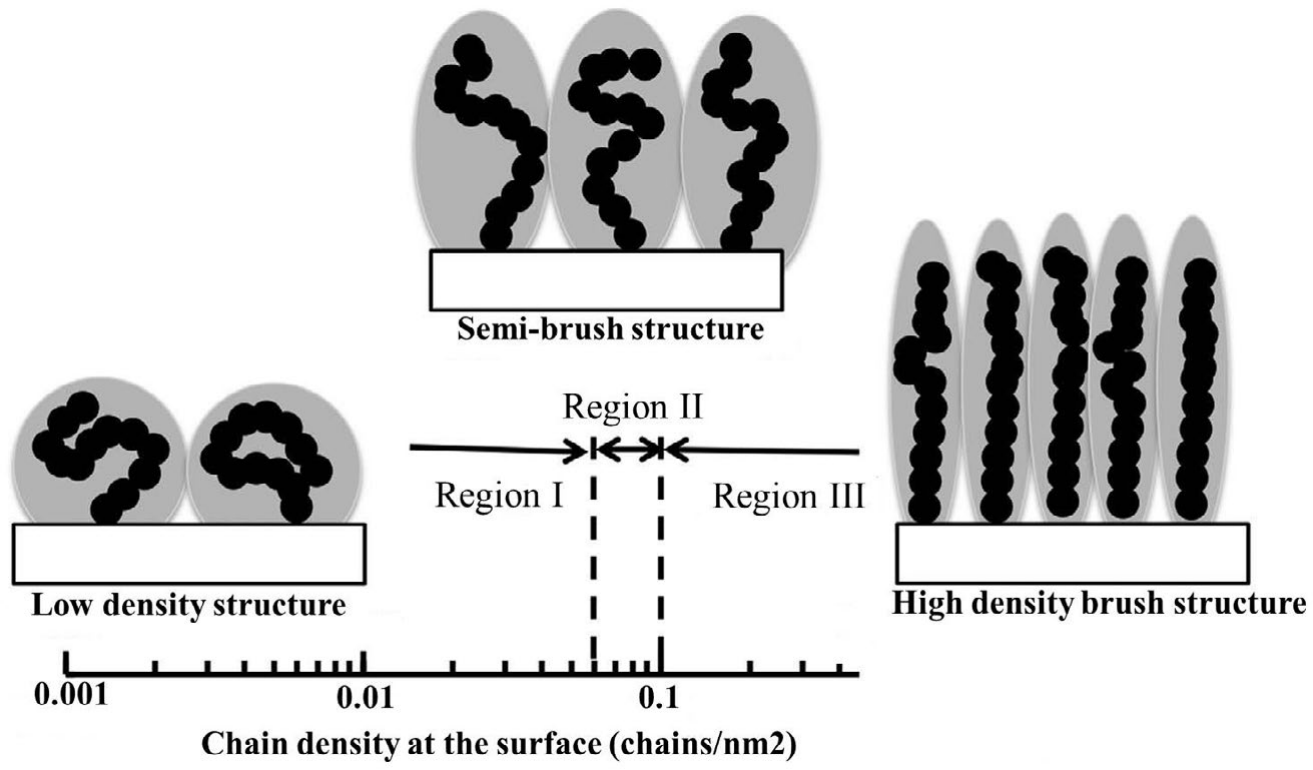
Graft conformation at various densities of polymer chains.[12]

This polymer grafted layer mimics the articular cartilage of actual physiological condition. Extremely hydrophilic polyelectrolyte brush surfaces exhibited high lubrication characteristics under water lubricant.[52,69] Two grafting methods are generally followed to graft the planar surfaces. The ‘grafting to’ approach could be used in physical polymer coating such as spin and dip coating. It promotes poor adhesion between the coated materials and the substrate. The coated materials may diffuse away in biological environments because the biological fluid acts as a good solvent of coated polymers. Conversely, ‘grafting from’ synthesizes high density polymer brushes those could change the conformation varying graft density in solvents.[70] Figure 6 shows the graft polymer conformation depends on the density of polymer chains.

The polymer structure seems to be mushroom-like at low graft density and it turns into a brush-like structure at high graft density. Such brush structure at high graft density are considerably thicker and range in size from a few nanometers to several micrometers. As a result, ‘grafting from’ layers are comparatively higher than ‘grafting to’ layers. Moro et al*.* [68] grafted the CLPE using poly(2-methacryloyloxyethyl phosphorylcholine) [MPC]) (PMPC) polymer. This PMPC-grafted surface reduced protein adsorption. It exhibited strong covalent bonding between PMPC and CLPE to improve weight bearing capacity. The lower friction coefficient and nearly zero wear rates were reported in recent studies of PMPC grafting on CLPE surfaces.[14,71,72]

#### 3.3.1. Recent studies of surface grafting for improved tribological performance

**Figure 7.**
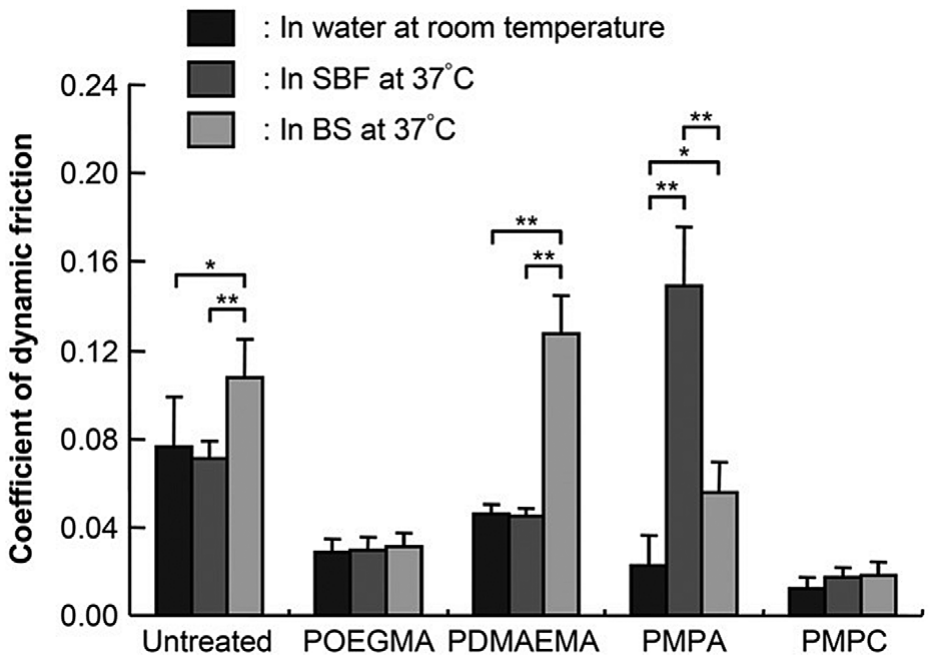
Coefficient of dynamic friction of polyelectrolyte-grafted CLPE samples under various lubrication conditions. Data are presented as means ± standard deviations. *Indicates *p* < 0.05, **indicates *p* < 0.01, and N.S. indicates no statistical difference.[12]

Surface grafting is a new technology developed to graft the implant surfaces in order to achieve high lubricity, low friction and wear. Few studies have been conducted on polymer grafting in the field of joint implants. Table 3 summarizes tribological studies of artificial implants over the last 10 years to identify the effect of surface grafting for improved friction and wear performance. A standard experimental set-up has been maintained in these studies, e.g. working load, contact pressure, frequency, testing lubricant, grafting technique, grafting thickness and type of the simulator. Different monomers such as OEGMA, DMAEMA and MPA are used to graft the implant surface. Kyomoto et al*.* [15] investigated the tribological performance of various polyelectrolyte-grafted CLPE samples under different lubrication conditions. Figure 7 summarizes the dynamic friction results of various polyelectrolyte-grafted CLPE samples.

**Table 2:** Summary of tribological studies on surface coating.




Abbreviations: TiN stands for titanium nitride, CVD stands for chemical vapour deposition, MW for microwave, PEEK for poly(etherether-ketone), RF for radio frequency and SBF for simulated body fluid.

**Table 3:** Summary of tribological studies on surface grafting.





Abbreviations: PDMAEMA stands for poly(2-(N,Ndimethylaminoethyl) methacrylate), POEGMA for poly(oligo(ethylene glycol) monomethacrylate) and PMPA for poly(2-(methacryloylethyl) phosphoric acid).

The polyelectrolyte-grafted CLPE samples exhibited lower friction coefficient than the untreated CLPE sample in all lubricants. No significant difference was observed between the lubricant for poly(oligo(ethylene glycol) monomethacrylate) (POEGMA)-grafted and PMPC-grafted surfaces where PMPC provided the highest lubricity. However, the poly(2-(N,N dimethylaminoethyl) methacrylate) (PDMAEMA)-grafted surface exhibited higher coefficient of friction in bovine serum (BS) lubricant compared to water and simulated body fluid (SBF) lubricants. Because the positively charged -NH^+^(CH_3_)_2_ group of PDMAEMA attracts negatively charged molecules in SBF it increases protein adsorption and resistance to motion.[77] Conversely, the negatively charged PMPA attracts the positively charged molecules and deters negatively charged molecules, resulting in the shrinkage or bridging of negatively charged polyelectrolyte chains in a solution of positively charged inorganic ions.[78] It reduces the mobility of polymer chains and increases the resistance to sliding motion. Figure 7 illustrates that PMPC-grafted CLPE exhibited the lowest friction coefficient in all lubrication conditions. The zwitterionic PMPC-grafted surface attracts the water molecules, and resists the protein molecules and the positively charged inorganic ions. As a result, it reduced protein adsorption as well as adhesive interaction between the implant interfaces.

Recently, Takatori et al*.* [16] investigated a biocompatible and highly hydrophilic surface via nanometre scaled grafting of PMPC onto CLPE. They found that PMPC-grafted surfaces captured water molecules and reduced the friction between the bearing surfaces via the hydration lubrication mechanism.[79] They revealed that a PMPC-grafted layer (100–150 nm in thickness) mimicking hydrogel structures of articular cartilage provided hydrophilicity and lubricity without affecting the CLPE substrate physical or mechanical properties. In addition, they reported that the PMPC-grafted surfaces were biologically inert and did not cause consequent bone-resorptive responses, indicating that this technique prevented wear particle production and biological reactions to such particles in total hip replacement (THR).[16]. Moro et al*.* [68] reported that PMPC-grafted CLPE surface significantly reduced the wear particle generation and the effect of PMPC grafting was maintained through 70 million cycles. Yarimitsu et al*.* [75] investigated the influence of dehydration and rehydration on the tribological performance of PMPC-grafted CLPE surface and found no significant effect on reducing friction coefficient. Similarly, vitamin E blending does not make any differences on friction results over the PMPC-grafted surface.[76] PMPC-grafted CLPE receiving gamma-ray irradiation showed higher ultimate tensile strength and elongation than those receiving non-extra or plasma irradiation.[72] Conversely, failure of PMPC-grafted CLPE with gamma-ray irradiation was significantly lower than those receiving non-extra or plasma irradiation. Moreover, plasma-irradiated PMPC-grafted CLPE showed significantly higher impact strength than those in the gamma-irradiated sample. Though PMPC-grafted CLPE liner treated with extra irradiation of plasma irradiation or gamma-ray exhibited no significant differences in wettability and wear resistance properties, the plasma irradiation showed improved oxidation resistance as compared to that treated with gamma-ray irradiation after accelerated ageing.[72]

## 4. Discussion

Surface modification techniques are used for tailoring surfaces to suit specific characteristics in the field of biomedical devices, microelectronic components, textile materials and food industry products. In this study, three types of surface modifications have been discussed. The study focused on the last 10 years of investigations to identify the status of hip implant research. Although there are many studies on joint prostheses, this paper critically reviews 27 experimental studies on hip implant studies. Any theoretical or numerical studies as well other joint prostheses related studies have been excluded to locate the limitations of currently developed surface modification techniques in the field of artificial hip joint prostheses. Moreover, a few studies were excluded due to insufficient data on tribological parameters. Friction and wear results of past studies are listed in Tables 1–3 to compare the tribological performances of different applied techniques on suitable implant surfaces. Deviations in experimental set-up and parameter selection have been observed over the studies. Hence, general comparisons between the three surface modification techniques were complex. Two major factors, friction coefficient and wear rate, are the evaluation criteria for tribological performances. The contact pressure depends on the applied load and the type of simulator. Pin-on-discs have higher contact pressure compared to hip simulators for the same applied load due to their point contact. The lubricants also play significant role on tribological behaviour of the modified surfaces.

**Figure 8.**
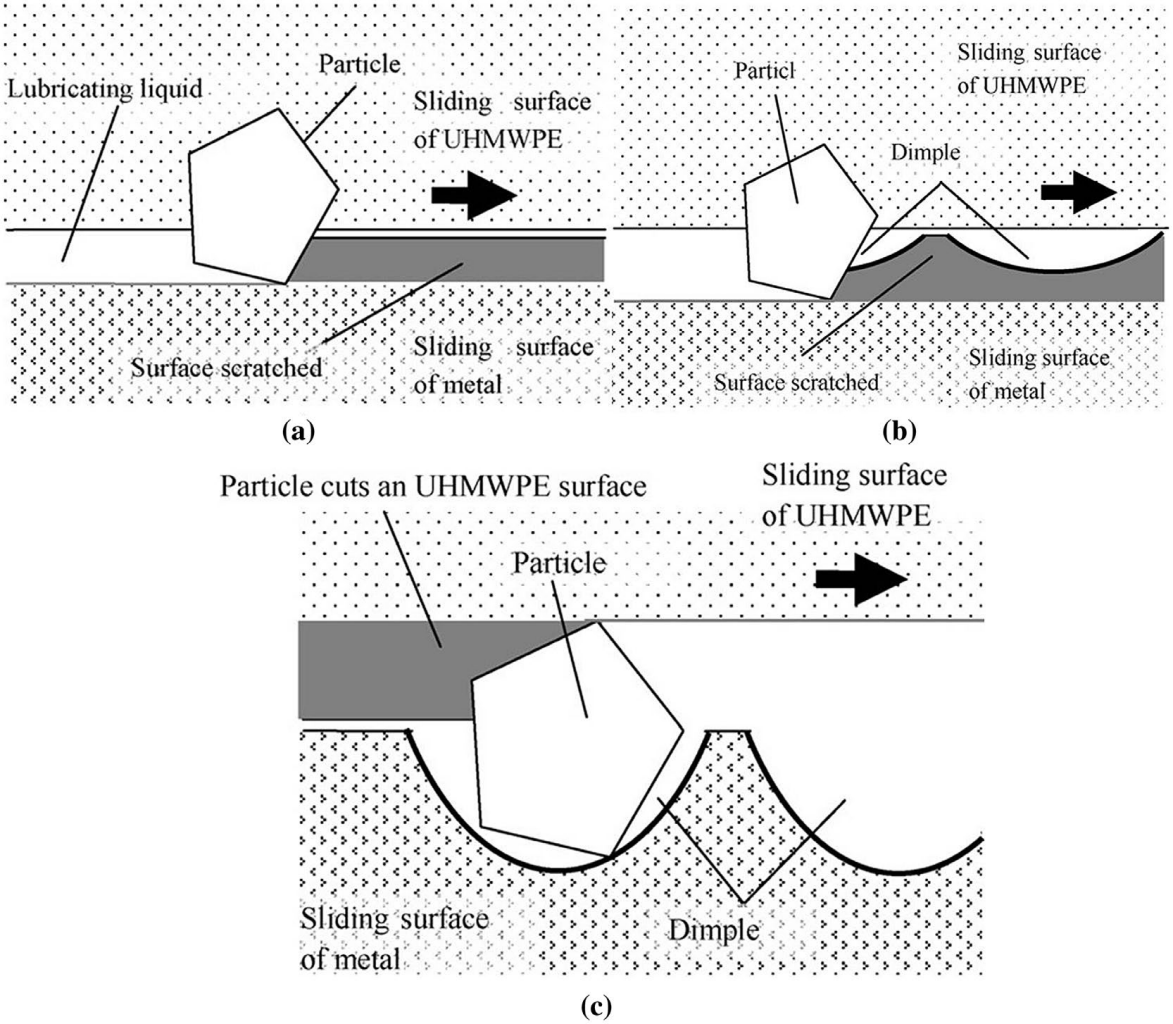
Wear mechanism for (a) without dimpled surfaces, (b) smaller depth dimpled surfaces and (c) suitable depth dimpled surfaces.[80]

Overall, the surface texturing technique exhibits significant reduction of friction coefficient for the implant surfaces. The wear rate is still significantly high for the textured surface. Figure 8 shows the wear mechanism of the implant surfaces in presence/ absence of dimples.

The wear debris generated due to sliding of contact interfaces leads to increased wear if the surface is not dimpled properly. Scratch marks shown in Figure 8(a) were due to the presence of wear particles on the contact interfaces and Figure 8(b) shows that dimple depth was not sufficient to trap the wear debris inside the dimples.[80] As a result, wear rate was increased with the sliding time as more debris was generated due to friction. Figure 8(c) shows that the suitable dimple depth trapped the wear particles properly, and thus reduced friction and wear of the contact interfaces.

Meng [81] reported that the microscale flow vortex existing in dimples developed hydrodynamic pressure that can release the entrapped wear debris from the dimples. The effect of vortex flow can be minimized by increasing dimple depth. Kai et al*.* [82] concluded that the effect of vortex flow becomes neutral at the deep zone of the high depth dimples. As a result, the particles velocity approached zero at the centre of the dimples. However, high depth of dimple could have negative effect on tribological outcomes. It could reduce the hydrodynamic pressure. Hence, the lubricating effect of the dimple would not be effective in this case.

**Figure 9.**
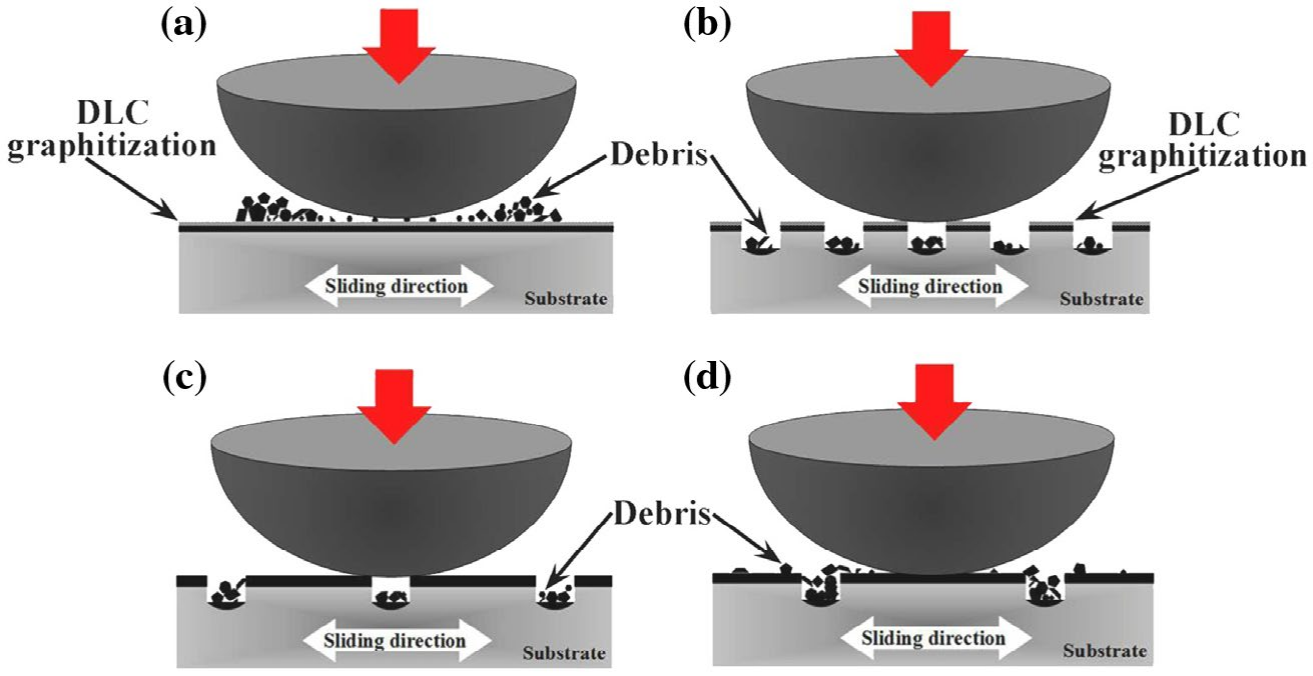
Cross-sectional schematic view of the wear mechanism model for (a) DLC-smooth, (b) DLC with higher density (c) DLC with suitable density (d) DLC with lower density.[43]

Involvement of surface coating with dimpled surfaces was also analysed. Continuous sliding motion between the contact interfaces generates considerable heat, which lowers the film hardness.[43] The generated wear particles also lower the graphitization temperature, resulting in adhesive wear by the graphitizing transformation. The smooth DLC coated surface directs to more abrasive wear (Figure 9(a)) that could be minimized with dimpled coated surface by trapping produced wear particles (Figure 9(b)). However, the dimples with higher densities reduce the contact area resulted high contact pressure.[58] Hence, the dimples with lower densities reduce the contact pressure as well as wear particle generation. Suitable dimples with lower density and sufficient depth could minimize the graphitization of coated surfaces (Figure 9(c) and (d)). The dimpled coated surface would be effective if the graphitization of the coated surface could be prevented and wear debris generation could be minimized. In spite of their significant improvement in friction results, further wear resistance is expected for longevity of the implant interfaces. Therefore, surface texturing and coating techniques are still under investigation to improve wear performance for implant applications. The PMPC-grafted layer has achieved a significant goal mimicking articular cartilage, which is very efficient in lubrication and has resulted in high wear resistant properties and desirable tribological outcomes. It exhibits high stability in oxidation, and therefore excellent mechanical properties for long-term hip bearings. Hence, the long-term clinical use of PMPC-grafted liner/head is now underway.

Recently Takatori et al*.* [16] reported the clinical success of PMPC grafter liner after seven years post-surgery. No osteolysis and revision surgery were reported and no adverse effect related to implanted liner was observed. However, cobalt–chromium (Co–Cr) or cobalt–chromium–molybdenum (Co-Cr-Mo) alloy was used as head material in recent studies against PMPC-grafted CLPE liner.[15,61,72,74,76] The metal ions, such as Cr^2+^, released from these types of orthopaedic implant, have been linked to an increased risk of cancer and allergic problems. Besides, weight gain is observed due to the absorption of water by CLPE material regardless of the grafting conditions.[61] Wear debris from CLPE materials initiates an inflammatory response followed by prosthetic loosening, and eventually causes osteolysis. Therefore, the material selection is crucial for ideal hip implantation. More research should be conducted using this surface modification technique for application on any type of polymeric materials, composites, metals and ceramics owing to better mechanical properties, lack of toxicity to the body, and minimal water absorption to the implant material. In most cases, a 26–28 mm diameter femoral head has been used. The larger diameter head with small radial clearance should be evaluated to identify the clinical utility of PMPC-grafted joint surfaces. Previous studies [10,14,61,74] only investigated friction behaviour, but film thickness measurement could reveal the actual lubrication conditions under different dynamic loading conditions. The film thickness for different lubricants, generated between head and cup materials under dynamic loadings, could be investigated. Further, the lubrication mechanism of polymer-grafted material could be investigated in presence of body oriented fluid resembling that of the physiological joint interface.

## 5. Summary and future work

A review of surface modification techniques on different implant surfaces has revealed that surface modifications have significant influence on enhanced tribological performances in varying applications and operating conditions. In the last couple of decades, substantial developments have been achieved from fundamental understanding, design of new and better implant surfaces, to both *in vivo* and *in vitro* applications. The study concludes the following:

1. Surface texturing techniques significantly reduce the friction coefficient by generating hydrodynamic pressure between the contact interfaces. However, the entrapped wear particles may be released from the dimples owing to the effect of associated vortex flow during hydrodynamic lift of working lubricant, and thus it could increase the wear particle generation. A suitable dimple depth with lower densities may lead to better tribological outcomes. Therefore, it is essential to develop theoretical models to optimize the surface geometry and to predict the lubrication mechanism depending on the operating conditions.
2. Surface coatings have solved the problems associated with adverse effect of metal bearings. They improve the mechanical properties of the modified surface as well as the wettability and wear resistance of implant surfaces. However, the generated heat lowers the graphitization temperature and, thus, results in earlier delamination of coated materials. Sometimes a slow crack advancement or interlayer dissolution causes delayed delamination in corrosive media. Poor adhesion of coated surfaces results in high wear rate and subsequently requires revision surgery. Therefore, further improvement is essential in surface coating technology choosing the proper interlayer to tailor the modified surface with specific characteristics.
3. A combination of surface texturing and coating techniques initially reduces friction and wear. But the continuously generated wear debris present on the contact area and the graphitization of the coated materials results in earlier failure of the modified surface maintaining enhanced tribological outcomes.
4. Interestingly, the PMPC-grafted surface layer is found to act as an efficient lubricant that can mimic the articular cartilage in physiological conditions. It exhibits significant reduction in friction coefficient than that of the textured or coated surfaces, and also provides almost zero wear rates in hip simulator studies. Recent studies also show the clinical success of PMPC-grafted surface.

Further investigations are required to identify the effect of PMPC surface grafting on different implant surfaces. The film formation results would establish better understanding of lubricating effect attributed to the PMPC-grafted surface. Therefore, a theoretical model needs to be developed considering the major parameters such as mechanical properties of material, lubricant viscosity, entraining velocity and applied load. The heat generation owing to sliding motion might have an impact on tribological behaviour and this influence needs to be investigated.

## Declaration of interests

The authors declare that there is no conflict of interests regarding the publication of this paper.
