# Corrigendum

**DOI:** 10.1080/14686996.2017.1338686

**Published:** 2017-06-19

Ghosh S, Abanteriba S. Status of surface modification techniques for artificial hip implants. Sci Tech Adv Mater. 2016;17:715—735. http://dx.doi.org/10.1080/14686996.2016.1240575

When the above article was published online, Fig. 1(a) was incorrectly sourced to ref. [33]. The correct reference number is [46]. Further, in Table 1, first row, the type of material should be SAE 52100 steel instead of Ti-6Al-4V alloy.

The authors apologize for this error.

